# Factors Associated with Regular Dental Checkups’ Discontinuation during the COVID-19 Pandemic: A Nationwide Cross-Sectional Web-Based Survey in Japan

**DOI:** 10.3390/ijerph19052917

**Published:** 2022-03-02

**Authors:** Katsuo Oshima, Hiroko Miura, Rumi Tano, Hideki Fukuda

**Affiliations:** 1 Department of Dental Technology, The Nippon Dental University College, Tokyo 102-8159, Japan; oshima@tky.ndu.ac.jp; 2 Division of Disease Control and Epidemiology, School of Dentistry, Health Sciences University of Hokkaido, Hokkaido 061-0293, Japan; 3 Department of Health Promotion, National Institute of Public Health, Saitama 351-0197, Japan; tano.r.aa@niph.go.jp; 4 National Institute of Public Health, Saitama 351-0197, Japan; fukuda.h.aa@niph.go.jp

**Keywords:** COVID-19 pandemic, regular dental checkup, dental visits, web-based survey, Japan

## Abstract

Managing oral health through regular dental checkups (RDCs) can help prevent dental diseases. Our study aimed to investigate the proportion and characteristics of those who stopped receiving RDCs owing to the coronavirus disease 2019 (COVID-19) pandemic. A nationwide web-based survey in Japan in September 2021 (3556 participants) revealed that 62.4% of participants had habitually received RDCs before COVID-19. Of these (*n* = 2219), 71.5% had received RDCs since the pandemic and 28.5% had not. Multiple logistic regression analysis revealed the following characteristics of those without RDCs since the pandemic: female (male, OR: 0.58, 95%CI: 0.45–0.74), lower household income (<2000 K JPY, OR: 1.45, 95%CI: 0.94–2.23; 2000 K–< 4000 K JPY, OR: 1.46, 95%CI: 1.08–1.98), fewer teeth (20–27, OR: 0.63, 95%CI: 0.39–1.03; ≥28, OR: 0.60, 95%CI: 0.36–0.98), and no interdental cleaning habits (OR: 0.51, 95%CI: 0.41–0.63). These results suggest that the disruption to RDCs owing to the pandemic is related to individual socioeconomic factors. Additionally, these individuals have poor oral health, which may be worsened by such barriers.

## 1. Introduction

Regular dental checkup (RDC) plays a pivotal role in maintaining and promoting oral health. Maintaining and managing oral health through RDCs can help prevent dental diseases such as dental caries and periodontal disease, and thus prevent tooth loss [1,2,3]. Preventing tooth loss and maintaining oral health can help enhance one’s quality of life [4,5,6]. Therefore, developing policies that establish systems in which a large number of people receive RDCs is very meaningful [7,8].

However, coronavirus disease 2019 (COVID-19) has spread worldwide since the first cases were reported in Wuhan, China, in 2019 [9]. In Japan, the first case of COVID-19 infection was confirmed on January 16, 2020, and the number of COVID-19 cases has been increasing since mid-March 2020 [10]. On March 11, 2020, the World Health Organization (WHO) declared COVID-19 a pandemic [11]. The pandemic restricted people’s mobility and affected various behaviors in their daily lives [12,13].

Moreover, several countries have reported that the COVID-19 pandemic has influenced dental visits. In a U.S. survey conducted between late May and early June 2020, 46.7% of the respondents reported delays in dental visits because of the COVID-19 pandemic, and of those who reported delays, 75% reported delays in receiving RDCs [14]. A study in Germany conducted in July 2020 found that 22% postponed dental visits because of the COVID-19 pandemic, and of those who reported postponement, 72% reported postponing RDCs [15]. In November 2020, a survey of workers in a local municipal office in Japan showed that 28.7% of the respondents stopped RDCs due to the COVID-19 pandemic [16]. In addition, at the beginning of the COVID-19 pandemic, urgent dental treatments, such as surgical procedures, were prioritized, while less urgent treatments, such as RDCs, were postponed [17,18,19].

These studies suggest that dental visits, particularly RDCs, have been disrupted by the COVID-19 pandemic. However, few studies have been conducted on a nationwide scale on whether people stopped receiving RDCs since the COVID-19 pandemic even if they had habitually received RDCs before the pandemic. Most previous studies were conducted in the early stages of the COVID-19 pandemic [14,15,16,17,18,19]. Although it has been more than 18 months since the WHO declared the COVID-19 pandemic, the situation of those who have not resumed RDCs is unclear.

As part of its countermeasures against COVID-19, the Japanese government declared a state of emergency in April 2020, which was implemented in each prefecture until September 2021 (as of December 2021) [20]. This declaration imposed restrictions on various activities such as events that attract numerous people, but not on visits to dental clinics. In February 2021, the president of the Japan Dental Association issued a statement that there are concerns regarding the occurrence and severity of dental caries and periodontal disease due to changes in lifestyle caused by the COVID-19 pandemic, and it is, therefore, advisable to have proper consultations with dentists to maintain oral health [21]. Hence, it is necessary to understand the actual situation of those who have stopped receiving RDCs since the COVID-19 pandemic on a national scale, and based on these data, policymakers should consider measures to safeguard oral health.

To assess the influence of the COVID-19 pandemic on RDCs, we conducted a nationwide web-based survey in Japan 18 months after the WHO declared the COVID-19 pandemic (September 2021). Using these data, we aimed to assess the number of people who have not had RDCs since the beginning of the pandemic even though they had habitually had RDCs before the pandemic. Thereafter, we analyzed the characteristics of those who stopped receiving RDCs since the COVID-19 pandemic.

## 2. Materials and Methods

### 2.1. Study Participants

This cross-sectional study used data from a web-based survey conducted over three days (6–8 September 2021). The survey was conducted by Macromill, Inc. (Tokyo, Japan) [22], a research company specializing in web-based surveys, with a registered pool of approximately 1.3 million people living in Japan. A total of 3556 survey participants of 20 years of age and above were randomly selected from the survey company’s database of registered individuals using the quota sampling method. The sample’s distribution across gender (male: 48.0%; female: 52.0%), age groups (20s: 12.1%; 30s: 15.0%; 40s: 17.7%; 50s: 14.9%; 60s: 17.4%; and 70s and above: 22.9%), and regional block (Hokkaido-region: 4.5%; Tohoku-region: 7.2%; Kanto-region: 33.7%; Chubu-region: 18.1%; Kinki-region: 16.2%; Chugoku-region: 5.9%; Shikoku-region: 3.2%; and Kyushu-region: 11.1%) was representative of the Japanese population. This distribution was based on statistics from the National Population Census of the Ministry of Internal Affairs and Communications [23].

The participants read an explanation of the purpose of the survey on the website and answered questions after agreeing to participate. They were given point-based incentives that could be converted to cash.

### 2.2. Survey Items

The survey items consisted of three major components: 1. changes in the receipt status of RDCs because of the COVID-19 pandemic; 2. individual socioeconomic factors; 3. oral health status. The survey forms were all prepared in Japanese.

1. We asked participants to select from the following four choices about changes in their receipt of RDCs before and after the COVID-19 pandemic: “I habitually received regular dental checkups before the COVID-19 pandemic, and I have received regular dental checkups since the pandemic”; “I habitually received regular dental checkups before the COVID-19 pandemic, but I have not received regular dental checkups since the pandemic”; “I did not habitually receive regular dental checkups before the COVID-19 pandemic, but I have received regular dental checkups since the pandemic”; “I did not habitually receive regular dental checkups before the COVID-19 pandemic, and I have not received regular dental checkups since the pandemic.” In this study, RDC was defined as “receiving at least one dental checkup during a year” [24].
2. Individual socioeconomic factors included gender, age, household income, employment status, marital status, and residential municipality. Participants’ ages were categorized into six groups: 20s, 30s, 40s, 50s, 60s, and 70 and above. The household income of the participants was categorized into six groups: <2000 K JPY, 2000 K–< 4000 K JPY, 4000 K–< 6000 K JPY, 6000 K–< 8000 K JPY, ≥8000 K JPY, and unknown. (As of 2018, the average annual household income in Japan was 5523 K JPY, and the median value was 4370 K JPY. 1 K JPY = USD 9.1, September 2021 [25].) The participants’ employment status was categorized into five groups: regular employees, self-employed, homemakers, nonregular employees, and not working. Participants’ marital status was categorized as married or single. The municipalities where the participants lived were categorized into four, based on the Japanese municipal system: metropolis (ordinance-designated cities with a population of 500,000 or more, and 23 special wards of Tokyo), core cities (ordinance-designated cities with a population of 200,000 or more, excluding metropolises), cities (cities with a population of 50,000 or more, excluding metropolises and core cities), and towns and villages (small municipalities that do not meet city requirements).
3. With regard to oral health status, we used data on the number of teeth and oral cleaning. The number of teeth was categorized into four groups: 0–9 teeth, 10–19 teeth, 20–27 teeth, and 28 or more teeth. Data on the number of teeth were obtained by questioning the participants. There are two ways to determine the number of teeth: a dentist’s examination or using a questionnaire, but there is no significant difference between the two methods [26,27]. Oral cleaning entails the number of times teeth are brushed per day and the habit of interdental cleaning.

### 2.3. Statistical Analysis

First, we assessed whether the participants were habituated to receiving RDCs before the COVID-19 pandemic. Among those who were habituated to receiving RDCs, we evaluated the proportion of those whose RDCs were interrupted since the COVID-19 pandemic.

Second, we stratified participants according to whether they had habitually received RDCs before the COVID-19 pandemic, and then evaluated the relationship between whether they received RDCs since the COVID-19 pandemic and their characteristics (individual socioeconomic factors and oral health status). The chi-square test was used to compare the proportions of the data (because “Number of teeth” and “Frequency of brushing teeth” are ordinal variables, the chi-square test for trend was used).

Third, to analyze the characteristics of those who have stopped receiving RDCs since the COVID-19 pandemic despite their habit of receiving RDCs before the pandemic, a multiple logistic regression analysis was conducted (those who have stopped receiving RDCs since the pandemic = 1, those who have received RDCs since the pandemic = 0). To analyze the primary outcome variable of the “those who have stopped receiving RDCs since the pandemic” group, explanatory variables of individual socioeconomic factors (gender, age, household income, employment status, marital status, and municipality of residence) and oral health status (the number of teeth and oral cleaning) were explored. In Model 1, individual socioeconomic factors were used as the explanatory variables. In Model 2, in addition to Model 1 factors, oral health status was entered as an explanatory variable. In both models, the odds ratio (OR) and 95% confidence interval (95% CI) of each explanatory variable were calculated using a forced entry model. The fit of each model was evaluated using significance tests. STATA version 14 (StataCorp LLC, College Station, TX, USA) was used for data management and statistical analysis. Statistical significance was set at *p* < 0.05, and *p* values between 0.05 and 0.10 were considered marginally significant.

### 2.4. Ethical Consideration

The survey participants were registered with Macromill, Inc. and agreed to their data being used for research. Furthermore, their personal information was protected by Macromill, Inc. This study was approved by the Research Ethics Committee of the School of Dentistry, Health Sciences University of Hokkaido, before conducting the study (July 2021, Approval Number: 213).

## 3. Results

### 3.1. Status of Participants Receiving RDCs Owing to the COVID-19 Pandemic

**Figure 1:**
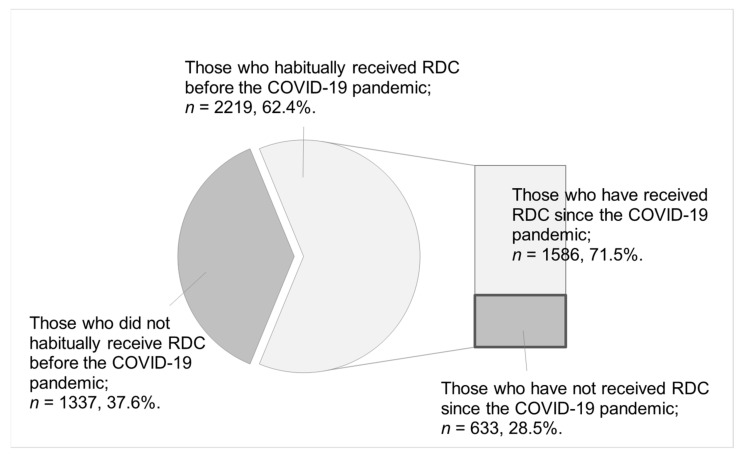
Status of participants receiving RDCs since the COVID-19 pandemic. Note. RDC = regular dental checkup; COVID-19 = coronavirus disease 2019.

Figure 1 shows the number and proportion of participants receiving RDCs before and since the COVID-19 pandemic. Of the 3556 participants, 62.4% habitually received RDCs before the pandemic, and 37.6% did not habitually receive RDCs. Of the 2219 participants who habitually received RDCs before the COVID-19 pandemic, 28.5% had not received RDCs since the pandemic.

### 3.2. Relationship between Receiving RDCs Owing to the COVID-19 Pandemic and the Study Participants’ Characteristics

The study participants were divided according to whether they had received RDCs before the COVID-19 pandemic; Table 1 shows the relationship between receiving RDCs since the pandemic and participants’ characteristics. Among those who habitually received RDCs before the COVID-19 pandemic, there were significant differences in gender (*p* < 0.001), age (*p* = 0.010), household income (*p* = 0.049), and habits of interdental cleaning (*p* < 0.001). Among those who did not habitually receive RDCs before the COVID-19 pandemic, there were significant differences in gender (*p* = 0.031), age (*p* = 0.032), and habits of interdental cleaning (*p* < 0.001).

### 3.3. Characteristics of Those Who Have Stopped Receiving RDCs since the COVID-19 Pandemic

Table 2 presents the odds ratios in the multiple logistic regression analysis for those who have not received RDCs since the COVID-19 pandemic despite habitually receiving RDCs before the pandemic, in comparison with those who have received RDCs, adjusted for their relationship to the participants’ characteristics (those who have stopped receiving RDC = 1, those who have received RDC = 0).

In the analysis of Model 1, characteristics of those who had not received RDCs since the COVID-19 pandemic were significantly higher among females (male, OR: 0.65, 95%CI: 0.51–0.82), in the younger age groups (20–29 years, OR: 1.55, 95%CI: 1.06–2.28), and lower household incomes (<2000 K JPY, OR: 1.54, 95%CI: 1.01–2.35; 2000 K–< 4000 K JPY, OR: 1.45, 95%CI: 1.08–1.96); there was a marginally significant association with living in towns and villages (towns and villages, OR: 1.46, 95%CI: 0.97–2.19), and with the age group of 50–59 years (OR: 1.33, 95%CI: 0.96–1.85).

In the analysis of Model 2, characteristics of those who had not received RDCs since the COVID-19 pandemic were significantly higher among females (male, OR: 0.58, 95%CI: 0.45–0.74), marginally significantly associated with the age groups of 20–29 years and 50–59 years (20–29 years, OR: 1.45, 95%CI: 0.98–2.14; 50–59 years, OR: 1.34, 95%CI: 0.96–1.87), significantly higher among those with lower household income (2000 K–< 4000 K JPY, OR: 1.46, 95%CI: 1.08–1.98), marginally significantly associated with lower household income (<2000 K JPY, OR: 1.45, 95%CI: 0.94–2.23), marginally significantly associated with living in towns and villages (towns and villages, OR: 1.49, 95%CI: 0.98–2.26), significantly higher in those with fewer teeth (≥28, OR: 0.60, 95%CI: 0.36–0.98), marginally significantly associated with fewer teeth (20–27, OR: 0.63, 95%CI: 0.39–1.03), and no habit of interdental cleaning (OR: 0.51, 95%CI: 0.41–0.63).

## 4. Discussion

### 4.1. Main Findings

In this study, using a nationwide web-based survey conducted in Japan in September 2021, we evaluated the proportion and characteristics of those who have stopped receiving RDCs since the COVID-19 pandemic despite habitually receiving it before. Our analysis showed that 62.4% of the 3556 participants habitually received RDCs before the COVID-19 pandemic. Of these participants (*n* = 2219), 28.5% had not received RDCs since the pandemic. Compared to those who have received RDCs since the pandemic, those who have not received RDCs were significantly more likely to be female, have lower household income, fewer teeth, and no habit of interdental cleaning. Therefore, our results suggest that there are several individual socioeconomic factors interrupting access to RDCs since the COVID-19 pandemic.

### 4.2. Influence on Receiving RDCs Due to the COVID-19 Pandemic and Implications of This Study

Previous studies have shown that since the beginning of the COVID-19 pandemic, RDCs have been postponed due to factors related to dental practices and patients [14,15,16,17,18,19]; these studies were conducted in the early stages of the pandemic. RDCs were postponed because of their low urgency among dental treatments [17,18,19], and many people feared dental visits due to the pandemic [28,29,30,31]. Therefore, the finding of previous studies that RDCs were delayed immediately after the COVID-19 pandemic is supported and should not be criticized.

However, our study was conducted in September 2021, that is, 18 months after the declaration of the COVID-19 pandemic. Our analysis showed that, among those with the habit of receiving RDCs before the COVID-19 pandemic, 28.5% stopped receiving RDCs since the pandemic. Our study was conducted at a different time period during the COVID-19 pandemic than previous studies, and hence cannot be compared to them [14,15,16,17,18,19]. While previous studies have shown the disruption in dental visits including RDCs, in the immediate aftermath of the COVID-19 pandemic [14,15,16,17,18,19], our study suggests that there is an association between barriers to continually receiving RDCs since the beginning of the COVID-19 pandemic and individual socioeconomic factors. In addition, continuing interruptions in receiving RDCs may lead to the deterioration of oral health. Thus, this study suggests the need for policy interventions to address the individual socioeconomic influences of the COVID-19 pandemic.

### 4.3. Relationship between Those Who Have Stopped Receiving RDCs since the COVID-19 Pandemic and Socioeconomic Factors

In the multiple logistic regression analysis results of Model 2 (Table 2), the socioeconomic factors of those who have stopped receiving RDCs reveal that RDC delays were higher among females and those with lower household income. In addition, there was a marginally significant association with those living in rural areas.

In several studies, the postponement of RDCs immediately after the COVID-19 pandemic was observed in more females than males [14,15]. Compared to males, females were reported to have more fears related to dental treatments during the COVID-19 pandemic [30]. Hence, prior studies support our findings that females are more likely to postpone receiving RDCs because of the COVID-19 pandemic.

Lower household income has been shown to have a significant impact on receiving RDCs [32]. In particular, the COVID-19 pandemic has had a major influence on people’s economic conditions [33]. Therefore, as demonstrated by this study, families with low household income have lowered the priority of RDCs in their household expenditure.

With regard to location, delays in dental visits owing to the COVID-19 pandemic were reported to be more common in urban than in rural areas [14]; areas with larger populations were more affected by people’s confusion, leading to delays in dental visits. In fact, it has been reported that more people in urban areas are infected with COVID-19 daily, than in rural areas [10]. However, in our study, rural areas were likelier than urban areas to have more interruptions in receiving RDCs after the pandemic. Takashima et al. [34] reported that those in rural areas tend to be more sensitive to COVID-19 prevention than those in urban areas because they fear criticism in addition to the fear of the COVID-19 infection. Therefore, those in rural areas possibly had a more negative perception of dental visits to receive RDCs than those in urban areas.

Of the socioeconomic factors that were relevant in our study, economic policy interventions, particularly with household income, can be introduced. In Japan, while various economic measures have been implemented since the COVID-19 pandemic, measures focusing on RDCs are necessary to safeguard oral health.

### 4.4. Relationship between Receiving RDCs and Oral Health Status

In the multiple logistic regression analysis results of Model 2 (Table 2), after adjusting for socioeconomic factors, many participants had few teeth, and many had no habit of interdental cleaning. Continuing to receive RDCs is an effective way to protect oral health [1,2,3,4,5,6]. In addition, it has been reported that there is an association between continuing to receive RDCs and socioeconomic factors [35]. Socioeconomic conditions exacerbated by the COVID-19 pandemic have worsened dental health [36]. In addition, those who discontinued regular dental visits during the COVID-19 pandemic because of concerns about dental visits had relatively poor periodontal health [16]. Our results suggest that those with poor oral health due to socioeconomic factors may have been further affected in the wake of the COVID-19 pandemic, interrupting the continual receipt of RDCs. The effect of the COVID-19 pandemic on household income is an obstacle to the continual receipt of RDCs. Hence, if the barriers to receiving RDCs are not overcome, it may lead to the further deterioration of oral health.

### 4.5. Limitations of This Study

This study had several limitations. First, we collected and analyzed the data using a web-based survey, and participants were limited to those registered with the web-based survey company. Although the rate of internet usage among Japanese people is increasing [37], those registered with web-based survey companies do not reflect the Japanese population; this may have led to a sample bias. Therefore, it cannot be denied that the proportion of the participants who were receiving RDCs may have also been affected by sample bias. Second, the survey participants were organized using the quota sampling method such that their distribution by gender, age groups, and regional block approximated that of the Japanese population. However, the samples of survey participants within each group (gender, age groups, and regional block) did not reflect the Japanese population. Third, this study was conducted as a cross-sectional survey spanning 18 months after the beginning of the COVID-19 pandemic. Therefore, it is unclear whether there is a causal relationship between those who have stopped receiving RDCs because of the pandemic and the associated factors in our analysis. Further detailed analyses are required on the status of the receipt of RDCs owing to the pandemic.

Despite these limitations, to the best of our knowledge, this study is the first to demonstrate on a national scale the characteristics of individuals who have stopped receiving RDCs since the COVID-19 pandemic despite habitually receiving it before. The COVID-19 pandemic is likely to be prolonged in the future, suggesting that this problem may occur not only in Japan but also in the oral health policies of other countries. Policymakers should be careful to maintain a system in which people receive equal access to RDCs, considering the socioeconomic conditions of individuals.

## 5. Conclusions

Using a nationwide web-based survey in Japan, we analyzed the proportion and characteristics of those who have stopped receiving RDCs since the COVID-19 pandemic despite their habitual engagement in RDCs before the pandemic. We found that 62.4% of the 3556 participants habitually received RDCs before the COVID-19 pandemic. Of these (*n* = 2219), 71.5% have received RDCs since the pandemic and 28.5% have not received RDCs. The characteristics of those who have not received RDCs since the COVID-19 pandemic, compared to those who have, include being female, having lower household income, fewer teeth, and no habit of interdental cleaning.

These results suggest that the disruption to RDCs since the beginning of the COVID-19 pandemic is related to the disparities in individual socioeconomic factors. In addition, individuals in such socioeconomic conditions have poor oral health, worsened by barriers to receiving RDCs.

## Figures and Tables

**Table 1:** Relationship between receiving RDCs owing to the COVID-19 pandemic and study participants’ characteristics.

	Total Number	Those Who Habitually Received RDC before the COVID-19 Pandemic (*n* = 2219)					Those Who Did Not Habitually Receive RDC before the COVID-19 Pandemic (*n* = 1337)				
Variable		Those Who Have Received RDC since the Pandemic		Those Who Have Not Received RDC since the Pandemic		*p* Value ^*^	Those Who Have Received RDC since the Pandemic		Those Who Have Not Received RDC since the Pandemic		*p* Value *
Total, *n* (%)	3556	1586	(71.5)	633	(28.5)		103	(7.7)	1234	(92.3)	
Gender, *n* (%)											
Male	1708	721	(75.9)	229	(24.1)	<0.001	48	(6.3)	710	(93.7)	0.031
Female	1848	865	(68.2)	404	(31.8)		55	(9.5)	524	(90.5)	
Age, *n* (%)											
20–29 years	430	123	(63.4)	71	(36.6)	0.010	28	(11.9)	208	(88.1)	0.032
30–39 years	535	201	(71.8)	79	(28.2)		21	(8.2)	234	(91.8)	
40–49 years	629	266	(72.5)	101	(27.5)		11	(4.2)	251	(95.8)	
50–59 years	529	215	(66.6)	108	(33.4)		14	(6.8)	192	(93.2)	
60–69 years	618	324	(76.1)	102	(23.9)		18	(9.4)	174	(90.6)	
≥70 years	815	457	(72.7)	172	(27.3)		11	(5.9)	175	(94.1)	
Household income, *n* (%)											
<2000 K JPY	299	119	(69.6)	52	(30.4)	0.049	9	(7.0)	119	(93.0)	0.412
2000 K–< 4000 K JPY	850	381	(70.7)	158	(29.3)		25	(8.0)	286	(92.0)	
4000 K–< 6000 K JPY	703	331	(77.0)	99	(23.0)		14	(5.1)	259	(94.9)	
6000 K–< 8000 K JPY	455	221	(72.2)	85	(27.8)		9	(6.0)	140	(94.0)	
≥8000 K JPY	478	243	(71.7)	96	(28.3)		14	(10.1)	125	(89.9)	
Unknown	771	291	(67.1)	143	(32.9)		32	(9.5)	305	(90.5)	
Employment status, *n* (%)											
Regular employee	1351	557	(71.9)	218	(28.1)	0.189	45	(7.8)	531	(92.2)	0.913
Non-regular employee	454	214	(73.5)	77	(26.5)		12	(7.4)	151	(92.6)	
Homemaker	756	381	(69.5)	167	(30.5)		19	(9.1)	189	(90.9)	
Self-employed and others	339	136	(66.3)	69	(33.7)		9	(6.7)	125	(93.3)	
Not working	656	298	(74.5)	102	(25.5)		18	(7.0)	238	(93.0)	
Marital status, *n* (%)											
Married	2296	1106	(72.3)	423	(27.7)	0.181	60	(7.8)	707	(92.2)	0.850
Single	1260	480	(69.6)	210	(30.4)		43	(7.5)	527	(92.5)	
Municipalities, *n* (%)											
Metropolis (pop 500,000+)	1242	583	(70.8)	240	(29.2)	0.239	37	(8.8)	382	(91.2)	0.101
Core cities (pop 200,000+)	685	310	(73.3)	113	(26.7)		11	(4.2)	251	(95.8)	
Cities (pop 50,000+)	1417	616	(72.2)	237	(27.8)		49	(8.7)	515	(91.3)	
Towns and villages	212	77	(64.2)	43	(35.8)		6	(6.5)	86	(93.5)	
Number of teeth, *n* (%)											
0–9	183	50	(61.7)	31	(38.3)	0.115 ^#^	5	(4.9)	97	(95.1)	0.645 ^#^
10–19	369	179	(69.6)	78	(30.4)		9	(8.0)	103	(92.0)	
20–27	1392	656	(72.2)	253	(27.8)		41	(8.5)	442	(91.5)	
≥28	1612	701	(72.1)	271	(27.9)		48	(7.5)	592	(92.5)	
Frequency of brushing teeth, *n* (%)											
≥three times daily	928	503	(73.3)	183	(26.7)	0.431 ^#^	24	(9.9)	218	(90.1)	0.008 ^#^
Twice daily	1894	826	(70.2)	350	(29.8)		61	(8.5)	657	(91.5)	
Once daily	674	249	(72.2)	96	(27.8)		17	(5.2)	312	(94.8)	
Sometimes/No brushing	60	8	(66.7)	4	(33.3)		1	(2.1)	47	(97.9)	
Interdental cleaning, *n* (%)											
Yes	2040	1199	(75.4)	392	(24.6)	<0.001	58	(12.9)	391	(87.1)	<0.001
No	1516	387	(61.6)	241	(38.4)		45	(5.1)	843	(94.9)	

Note. RDC = regular dental checkup, * Pearson’s chi-square test, ^#^ chi-square test for trend.

**Table 2:** Characteristics of those who have not received RDCs since the COVID-19 pandemic, despite habitually receiving RDCs before the pandemic.

	Model 1			Model 2		
Variable	OR	95%CI	*p* Value	OR	95%CI	*p* Value
Gender						
Male	0.65	(0.51–0.82)	<0.001	0.58	(0.45–0.74)	<0.001
Female	1.00	Reference		1.00	Reference	
Age						
20–29 years	1.55	(1.06–2.28)	0.025	1.45	(0.98–2.14)	0.064
30–39 years	1.04	(0.73–1.48)	0.810	1.04	(0.73–1.48)	0.838
40–49 years	1.00	Reference		1.00		
50–59 years	1.33	(0.96–1.85)	0.091	1.34	(0.96–1.87)	0.088
60–69 years	0.83	(0.59–1.15)	0.266	0.85	(0.60–1.19)	0.332
≥70 years	0.95	(0.69–1.32)	0.757	0.94	(0.67–1.32)	0.710
Household income						
<2000 K JPY	1.54	(1.01–2.35)	0.045	1.45	(0.94–2.23)	0.089
2000 K–< 4000 K JPY	1.45	(1.08–1.96)	0.014	1.46	(1.08–1.98)	0.014
4000 K–< 6000 K JPY	1.00	Reference		1.00	Reference	
6000 K–< 8000 K JPY	1.29	(0.91–1.81)	0.147	1.28	(0.91–1.82)	0.157
≥8000 K JPY	1.31	(0.94–1.83)	0.116	1.32	(0.94–1.86)	0.106
Unknown	1.53	(1.12–2.09)	0.007	1.53	(1.12–2.09)	0.008
Employment status						
Regular employee	1.00	Reference		1.00	Reference	
Non-regular employee	0.76	(0.55–1.06)	0.111	0.76	(0.54–1.07)	0.112
Homemaker	0.96	(0.70–1.32)	0.785	0.96	(0.70–1.33)	0.820
Self-employed and others	1.34	(0.94–1.90)	0.102	1.33	(0.93–1.89)	0.119
Not working	0.92	(0.66–1.28)	0.625	0.96	(0.68–1.34)	0.804
Marital status						
Married	1.00	(0.80–1.26)	0.982	1.03	(0.82–1.30)	0.773
Single	1.00	Reference		1.00	Reference	
Municipalities						
Metropolis (pop. 500,000+)	1.06	(0.85–1.31)	0.613	1.13	(0.91–1.41)	0.265
Core cities (pop. 200,000+)	0.96	(0.73–1.25)	0.755	0.99	(0.76–1.30)	0.939
Cities (pop. 50,000+)	1.00	Reference		1.00	Reference	
Towns and villages	1.46	(0.97–2.19)	0.070	1.49	(0.98–2.26)	0.059
Number of teeth						
0–9				1.00	Reference	
10–19				0.74	(0.43–1.27)	0.269
20–27				0.63	(0.39–1.03)	0.066
≥28				0.60	(0.36–0.98)	0.040
Frequency of brushing teeth						
≥three times daily				0.97	(0.71–1.32)	0.838
Twice daily				1.13	(0.86–1.50)	0.387
Once daily				1.00	Reference	
Sometimes/No brushing				1.54	(0.43–5.55)	0.513
Interdental cleaning						
Yes				0.51	(0.41–0.63)	<0.001
No				1.00	Reference	

Note. RDC = regular dental checkup; OR = odds ratio; 95%CI, 95% confidence interval. Model 1: Number of observations = 2219, χ^2^_(19)_ = 52.39, Log likelihood = −1300.45, *p* < 0.001; Model 2: Number of observations = 2218, χ^2^_(26)_ = 103.37, Log likelihood = −1274.62, *p* < 0.001.

## Data Availability

Data cannot be shared publicly, because no informed consent was given by the participants for open data sharing.
